# Applications and prospects of genome editing in plant fatty acid and triacylglycerol biosynthesis

**DOI:** 10.3389/fpls.2022.969844

**Published:** 2022-08-31

**Authors:** Mid-Eum Park, Hyun Uk Kim

**Affiliations:** ^1^Department of Molecular Biology, Sejong University, Seoul, South Korea; ^2^Department of Bioindustry and Bioresource Engineering, Plant Engineering Research Institute, Sejong University, Seoul, South Korea

**Keywords:** acyltransferase, CRISPR/Cas9, *FAD2*, *FAE1*, *FATB*, *KASI*, lipase, TAG

## Abstract

Triacylglycerol (TAG), which is a neutral lipid, has a structure in which three molecules of fatty acid (FA) are ester-bonded to one molecule of glycerol. TAG is important energy source for seed germination and seedling development in plants. Depending on the FA composition of the TAG, it is used as an edible oil or industrial material for cosmetics, soap, and lubricant. As the demand for plant oil is rising worldwide, either the type of FA must be changed or the total oil content of various plants must be increased. In this review, we discuss the regulation of FA metabolism by Clustered regularly interspaced short palindromic repeats (CRISPR)/Cas9, a recent genome-editing technology applicable to various plants. The development of plants with higher levels of oleic acid or lower levels of very long-chain fatty acids (VLCFAs) in seeds are discussed. In addition, the current status of research on acyltransferases, phospholipases, TAG lipases, and TAG synthesis in vegetative tissues is described. Finally, strategies for the application of CRISPR/Cas9 in lipid metabolism studies are mentioned.

## Introduction

**Figure 1:**
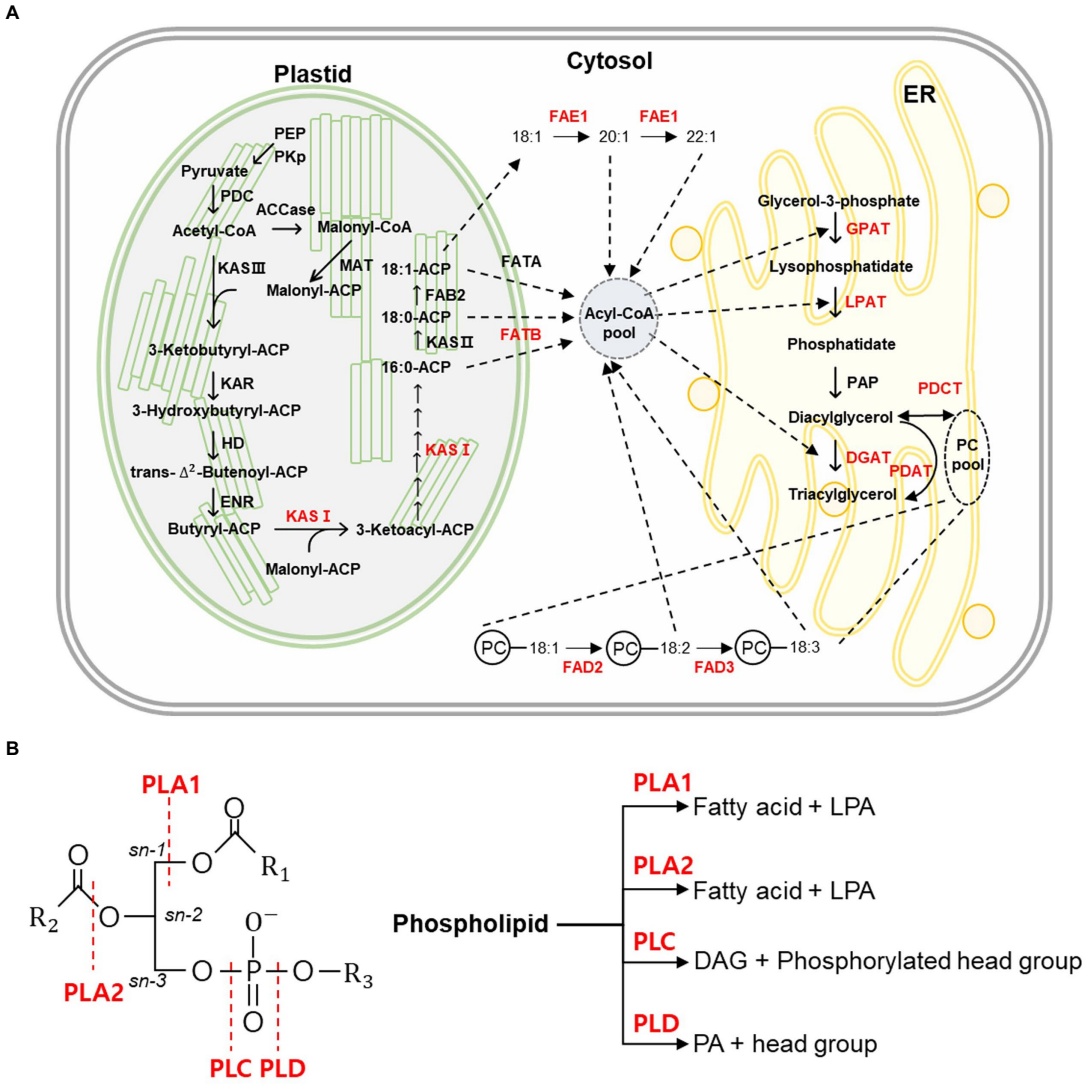
Fatty acid, triacylglycerol synthesis pathway, and function of phospholipase. **(A)** A schematic diagram of fatty acid and triacylglycerol synthesis pathway in plants. The figure illustrates the acyl-CoA synthesis pathway in the plastid and triacylglycerol (TAG) synthesis pathway by acyltransferase in endoplasmic reticulum (ER). Polyunsaturated fatty acids are synthesized in phosphatidylcholine (PC) by desaturase enzymes such as fatty acid desaturase 2 (FAD2) and FAD3. The FAE1 enzyme elongates the 18:1 fatty acid to 20:1 or 22:1, which are very long-chain fatty acids. Red-colored letters indicate the enzyme that was studied using Clustered regularly interspaced short palindromic repeats (CRISPR) and CRISPR-associated protein 9 (CRISPR/Cas9). The dotted lines represent the flow of the fatty acids in fatty acid and triacylglycerol synthesis. ACCase, acetyl-CoA carboxylase; ACP, acyl carrier protein; CoA, coenzyme A; DGAT, diacylglycerol acyltransferases; ENR, enoyl-ACP reductase; ER, endoplasmic reticulum; FAB2, fatty acid biosynthesis 2; FAD2, fatty acid desaturase 2; FAD3, fatty acid desaturase 3; FAE1, fatty acid elongase 1; FATA, fatty acyl-ACP thioesterase A; FATB, fatty acyl-ACP thioesterase B; GPAT, glycerol-3-phosphate acyltransferase; HD, 3-hydroxy acyl-ACP dehydratase; KAR, 3-ketoacyl-ACP reductase; KAS, β-ketoacyl-acyl carrier protein synthase; LPAT, lysophosphatidic acid acyltransferase; MAT, malonyl-CoA/ACP transacylase; PAP, phosphatidate phosphatase; PC, phosphatidylcholine; PDAT, phospholipid:diacylglycerol acyltransferase; PDC, pyruvate dehydrogenase complex; PDCT, phosphatidylcholine:diacylglycerol cholinephosphotransferase; PEP, phosphoenolpyruvate; and PKp, Plastidial pyruvate kinase. **(B)** The reaction of phospholipase in plants. Plants have four different forms of phospholipases (PLA1, PLA2, PLC, and PLD). Phospholipase is the enzyme that hydrolyzes phospholipids. The cleavage site of phospholipase is shown on the left figure and indicated by the red dotted lines. The right figure shows the product produced by phospholipase. DAG, diacylglycerol; LPA, lysophosphatidate; PA, phosphatidate; PLA1, phospholipase A1; PLA2, phospholipase A2; PLC, phospholipase C; and PLD, phospholipase D.

Fatty acids (FAs) are synthesized by the addition of two carbons by fatty acid synthase (FAS) in the plastid (Rawsthorne, 2002). FA biosynthesis is initiated by acetyl-CoA carboxylase, which converts acetyl-CoA to malonyl-CoA (Sasaki and Nagano, 2004). Malonyl-CoA is converted to malonyl-ACP by malonyl-CoA: acyl carrier protein (ACP) transacylase (Lessire and Stumpe, 1983). Malonyl-ACP is combined with acetyl-CoA by *β*-ketoacyl-acyl carrier protein synthase III (KAS III) to synthesize 4:0-ACP (Clough et al., 1992). KAS I is involved in the elongation from 4:0-ACP to 16:0-ACP and is synthesized as 18:0-ACP by KAS II (Shimakata and Stumpf, 1982). The 18:0-ACP is desaturated to 18:1-ACP by fatty acid biosynthesis 2 (FAB2; Lightner et al., 1994). Free FAs are removed from ACP by fatty acyl-ACP thioesterase A (FATA) and fatty acyl-ACP thioesterase B (FATB) and exit the plastid to form an acyl-CoA pool in the cytoplasm (Jones et al., 1995; Salas and Ohlrogge, 2002). Subsequently, acyl-CoAs are sequentially transferred to glycerol-3-phosphate (G3P) by acyltransferase enzymes in the endoplasmic reticulum (ER) to form triacylglycerol (TAG; Li-Beisson et al., 2013). To synthesize TAG, lysophosphatidic acid (LPA) is formed by attaching acyl-CoA at the *sn-1* position of the G3P backbone by glycerol-3-phosphate acyltransferase (GPAT; Shockey et al., 2016). Lysophosphatidic acid acyltransferase (LPAT) then transfers acyl-CoA to the *sn-2* position of LPA to form phosphatidic acid (PA). Phosphate at the *sn-3* position of PA is cleaved by phosphatidate phosphatase (PAP) to form diacylglycerol (DAG; Carman and Han, 2006). Finally, TAG is produced by attaching acyl-CoA to the *sn-3* position of DAG using diacylglycerol acyltransferase (DGAT; Cases et al., 1998; Zou et al., 1999; Figure 1).

Polyunsaturated fatty acids (PUFAs) present in TAG are synthesized in phosphatidylcholine (PC), a membrane lipid (He et al., 2020). First, the oleic acid (18:1) of *sn-2* in PC is converted to linoleic acid (18:2) by fatty acid desaturase 2 (FAD2), and then, linoleic acid (18:2) is converted to linolenic acid (18:3) by FAD3 (Lemieux et al., 1990; Browse et al., 1993; Dar et al., 2017). PUFAs are released from PC to form an acyl-CoA pool by reverse reaction of LPCAT (Lager et al., 2013). These acyl-CoAs are transferred into TAG through the acyl-CoA-dependent pathway by ER acyltransferases, as discussed above (Zou et al., 1999; Kim et al., 2005; Shockey et al., 2016). In addition, an acyl-CoA-independent pathway can directly synthesize TAG by transferring PUFAs in PC to DAG by phospholipid:diacylglycerol acyltransferase (PDAT; Dahlqvist et al., 2000; Figure 1A).

The discovery and functional studies of genes related to FA and TAG synthesis were carried out by forward genetics using mutants induced by ethyl methanesulfonate (EMS), and reverse genetics using T-DNA insertion mutants in *Arabidopsis thaliana*, a model plant (Lemieux et al., 1990; Browse et al., 1993; Lightner et al., 1994; Mcconn et al., 1994; Wu et al., 1994). Genetic studies on lipid metabolism in various crops have been conducted based on the insights from studies on *Arabidopsis* (Li-Beisson et al., 2013).

In most crops, FA composition consists of five common FAs: 16:0, 18:0, 18:1, 18:2, and 18:3 (Buchanan et al., 2015). However, some wild plants have unusual FAs (e.g., *ω*-hydroxy, 9,10-epoxy, caprylic acid, and ricinoleic acid) with specific functional groups on the FA carbon chain (Cahoon and Li-Beisson, 2020). Unusual FAs present in wild plants are industrially useful because they serve as raw materials for various polymers produced by chemical processes (Cahoon and Li-Beisson, 2020). Common FAs present in crops can also be useful in the food industry if the proportion of single types of FAs increases. For instance, vegetable oil with increased oleic acid content is suitable for frying and cooking oils (Przybylski and Aladedunye, 2012). In plant lipid metabolism engineering, strategies have mainly been used to control the FA pathway by overexpressing or mutating a specific gene to eliminate its function (Napier et al., 2014; Haslam et al., 2016). Clustered regularly interspaced short palindromic repeats (CRISPR) and CRISPR-associated protein 9 (CRISPR/Cas9), a recently emerged gene-editing tool, can easily and quickly edit the genome by precisely targeting a gene. Compared with traditional breeding process which requires removal of unfavored traits through repeated backcrossing and selection (Chen et al., 2019), CRISPR/Cas9 technology allows rapid development of a new cultivar with desirable traits. Besides, the conventional EMS mutagenesis is being replaced by CRISPR/Cas9 method because of its precision and completeness of mutation. In this review, we summarize the studies of CRISPR/Cas9-based knockout of mutants involved in lipid metabolism and discuss future directions in implementing this technology for the development of new oilseed crops.

## CRISPR/Cas9 and lipid metabolic engineering

Transcription activator-like effector nucleases (TALENs), zinc-finger nucleases (ZFNs), and CRISPR/Cas9 enable researchers to edit the genome in plants (Durai et al., 2005; Jinek et al., 2012; Joung and Sander, 2013). In TALENs, the Tal effector recognizes the DNA sequence and the FokI endonuclease cuts the DNA (Joung and Sander, 2013). In ZFNs, the Fok I endonuclease cuts DNA, however, unlike for TALEN, the zinc finger domain recognizes the DNA sequence (Durai et al., 2005). Both systems are widely used for genome editing; however, they are difficult to handle and require a long time for application to organisms compared to CRISPR/Cas9 (Chandrasegaran and Carroll, 2016). In contrast, CRISPR/Cas9 is less expensive and easy to use for any organism (Jinek et al., 2012; Chandrasegaran and Carroll, 2016).

CRISPR/Cas9 was first identified in the bacterial immune system (Brouns et al., 2008). The CRISPR/Cas9 system consists of two parts: a guide RNA (gRNA) and Cas9 protein. The gRNA has two parts: crispr RNA (crRNA) is a complementary sequence to the target gene, and trans-activating crispr RNA (tracrRNA) serves as a scaffold for linking with the Cas9 protein (Jinek et al., 2012). crRNA and tracrRNA are collectively called gRNA (Jinek et al., 2012; Ran et al., 2013). The Cas9 protein cuts double-stranded DNA, causing a double-strand break (DSB; Ran et al., 2013). The most used Cas9 protein is *Streptococcus pyogenes* Cas9 (SpCas9), which cuts the 3 bp position in front of the protospacer adjacent motif (PAM) corresponding to the NGG sequence in the DNA (Ran et al., 2013). Depending on the type of Cas9, Cas9 recognizes a PAM sequence that is different from NGG (Leenay and Beisel, 2017). Since the introduction of CRISPR/Cas9 technology, genome editing research has been conducted in various organisms, such as plants, humans, and microalgae (Jeon et al., 2017; Jaganathan et al., 2018; Subedi et al., 2020; Li et al., 2020b).

**Figure 2:**
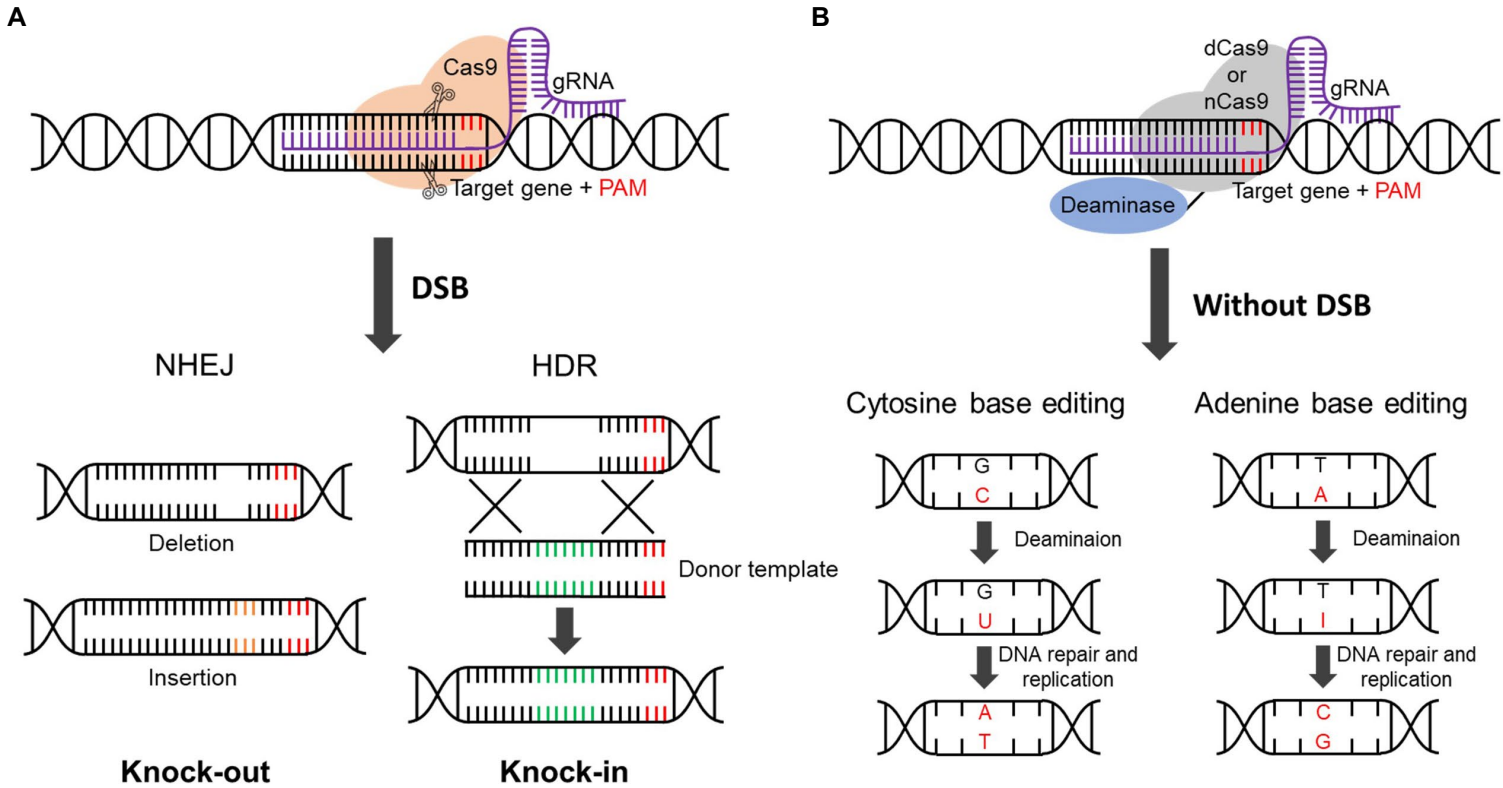
CRISPR/Cas9 and base editing mechanism. **(A)** Repair mechanism of CRISPR/Cas9. CRISPR/Cas9 is composed of Cas9 protein and gRNA. Cas9 recognizes the PAM sequence and cleaves 3 bp upstream of PAM to cause the DSB. When a DSB occurs, the cellular repair mechanism, including NHEJ and HDR processes, is initiated. NHEJ causes deletion or insertion, resulting in gene knockout due to a frameshift change. The donor template is inserted by HDR, and knockin occurs. DSB, Double-strand break; NHEJ, non-homologous end joining; HDR, homology-directed repair; PAM, protospacer adjacent motif. **(B)** Mechanism of base editing. The cytosine base editor or adenine base editor is made up of dead Cas9 (dCas9) or nickase Cas9 (nCas9) fused with cytosine deaminase or adenine deaminase. Cytosine deaminase removes the amine group from cytosine, resulting in a U-G mismatch. The U-G pair is converted to T-A by DNA repair and DNA replication. Adenine deaminase converts adenine to inosine by removing the amine group, resulting in an I-T mismatch. The I-T pair is converted to G-C by DNA repair and DNA replication.

The mechanism of CRISPR/Cas9 involves the formation of DSB by Cas9, which leads to two DNA repair mechanisms: non-homologous end-joining (NHEJ) and homology-directed repair (HDR; Cong et al., 2013; Ran et al., 2013; Sander and Joung, 2014; Figure 2A). As the NHEJ process directly repairs through insertion or deletion, gene knockout follows where DSB has occurred (Jiang and Doudna, 2017). Compared to NHEJ, HDR leads to knock-in, in which a DNA fragment is inserted into the DSB region (Jiang and Doudna, 2017).

CRISPR/Cas9-based technology for editing specific nucleotide sequence has also emerged recently. Dead Cas9 (dCas9) or nickase Cas9 (nCas9) fused with cytosine deaminase or adenine deaminase can convert specific nucleotides (C–T or A–G) without any DNA cleavage, it is referred to as the cytosine base editor or adenine base editor (Figure 2B; Komor et al., 2016; Nishida et al., 2016; Gaudelli et al., 2017). If one amino acid needs to be changed rather than knocked out, the base editor can be a powerful tool. For example, rice (*Oryza sativa*) has been modified to be herbicide resistant plant through base editing in the *acetyl CoA carboxylase* (*ACCase*) gene (Liu et al., 2020). A strategy for base editing technology in oil palm is also being introduced (Yarra et al., 2020). In addition to Cas9 and deaminase types, an online tool to design gRNA, and analysis methods to confirm mutation pattern are discussed (Yarra et al., 2020). As CRISPR/Cas9 technology develops rapidly, it has become easier and faster to knock out genes. One or two gRNA are generally used to generate single-gene mutants (Cong et al., 2013; Wang et al., 2015). More than two guide RNAs can be designed using cellular tRNA processing to target multiple genes (Xie et al., 2015). In plants, to avoid issues related to GMOs, the gene is mutated by directly injecting Cas9 protein and gRNA into the plant protoplast rather than introducing the *Agrobacterium* plasmid vector (Woo et al., 2015). The transient expression of Cas9 is also good strategy to develop transgene-free mutants because Cas9 DNA or RNA is degraded (Zhang et al., 2016). Currently, plant lipid metabolic engineering using CRISPR/Cas9 involves reduction of PUFAs that cause rancidity in oil, while increasing monounsaturated fatty acids (MUFAs) and inhibiting the synthesis of unhealthy saturated fatty acids (SFAs) and very long-chain fatty acids (VLCFAs).

### Mutation in *FAD2*

Polyunsaturated fatty acids (18:2 and 18:3) were synthesized from PC in the ER (Ohlrogge and Browse, 1995). In PUFA synthesis, FAD2 desaturases 18:1 at the *sn-2* position of PC into 18:2, and FAD3 desaturates from 18:2 to 18:3 (Lemieux et al., 1990; Browse et al., 1993; Dar et al., 2017; Figure 1A). As PUFAs are essential nutrients in humans, they can be beneficial to human health (Lee et al., 2016). However, it is easily oxidized at room temperature, and trans-fats are formed during deep-fat frying at high temperatures, they are unsuitable for salad dressings or cooking oils (Przybylski and Aladedunye, 2012; Saini and Keum, 2018). Therefore, making oil crops high in oleic acid is important in the food industry. Among oil crops, grape seed, sunflower, cotton, corn, soybean, camelina, perilla, and linseed have a high proportion of PUFAs in the oils (Dubois et al., 2007).

The function of FAD2 was first identified in the EMS and T-DNA mutants of *Arabidopsis* (Lemieux et al., 1990; Okuley et al., 1994). When FAD2 loses its activity, the PUFA content of the seed oil decreases, and the oleic acid content increases; however, it is sensitive to salt stress during seed germination and seedling growth (Zhang et al., 2012). Recently, various *fad2* alleles have been reported to weaken the function of FAD2 through base editing, increase oleic acid content, and confer resistance to salt stress (Park et al., 2021).

Studies have reported the elimination of the FAD2 function using CRISPR/Cas9 in various crops (Table 1). In tobacco (*Nicotiana tabacum* L.), two homozygous *ntfad2-2* mutants were found and their FA composition was checked. Consequently, the oleic acid content increased from 12 to 79% in mutants (Tian et al., 2020a). In rapeseed (*Brassica napus*), the *FAD2* gene was knocked out using CRISPR/Cas9 in two cultivars (Okuzaki et al., 2018; Huang et al., 2020). First, compared with the wild type, which produces 74% oleic acid, *BnFAD2_Aa* of the cultivar Westar was knocked out to increase the oleic acid content to 80% (Okuzaki et al., 2018). Second, oleic acid of each knockout mutant of *BnFAD2_A5* and *BnaFAD2_C5* in cultivar J9707 was enhanced to 73–82%, whereas oleic acid was 66% in the wild type (Huang et al., 2020). *FAD2* gene knockout studies of soybean (*Glycine max*) have been performed by several groups. In the cultivar Jinong38 (JN38), oleic acid content was 45–65% when *GmFAD2-2* was knocked out (Al Amin et al., 2019). Oleic acid content increased to 34.47 and 40.45% in *GmFAD2-1A* and *GmFAD2-2A* mutants, respectively, and double mutants of *GmFAD2-1A* and *GmFAD2-2A* induced a high oleic acid content of up to 72% (Wu et al., 2020). In another group, they targeted both *GmFAD2-1A* and *GmFAD2-1B* in order to create double knockout mutants in the cultivar Maverick, and the oleic acid content was dramatically increased to 80% (Do et al., 2019).

**Table 1:** Decrease in polyunsaturated fatty acid by CRISPR/Cas9.

**Gene name**	**Technique**	**Promoter of Cas9**	**Method**	**Phenotype**	**Oleic acid WT (%)**	**Oleic acid Mutant (%)**	**Mutation type**	**References**
*AhFAD2A* *AhFAD2B*	CRISPR/Cas9	CamV 35S	Hairy root transformation	-	36 ~ 67%	Not harvest the seeds	G448A (*ahFAD2A*), +1 bp, and G451T (*ahFAD2B*)	Yuan et al., 2019
*NtFAD2-2*	CRISPR/Cas9	CamV 35S	*Agrobacterium*-mediated transformation	No side effect	~12%	79%	-1 and −5 bp	Tian et al., 2020a
*AtFAD2*	Base editing	RPS5A (*Arabidopsis*)	Floral dipping	Resistance to salt stress	18.5%	57.9%	A295G, D298E	Park et al., 2021
						64.7%	A295V, T296M	
						30.6%	A295V	
						29.6%	A295G	
	CRISPR/Cas9	CamV 35S	Floral dipping	-	16.2%	~59.8%	+1 bp	Jiang et al., 2017
*BnFAD2*	CRISPR/Cas9	Ubiquitin4-2 (*Petroselinum crispum*)	*Agrobacterium*-mediated transformation	No difference	cv. Westar(74.6%)	*BnFAD2_Aa* (80%)	-4 bp	Okuzaki et al., 2018
		Ubiquitin (rice)			cv. J9707 (66.7%)	*BnaFAD2.A5* (73.1–82.3%)	−1bp, −1bp and S1, −2bp, −13bp, −80bp, +1bp, +1bp and +1bp, +1bp and −2bp, +1bp and −7bp	Huang et al., 2020
						*BnaFAD2.C5* (73–74%)	−3 and +1 bp	
*CsFAD2*	CRISPR/Cas9	Ubiquitin4-2 (*Petroselinum crispum*)	Floral dipping	All *CsFAD2* gene mutants → slow growth, twisted leaves, delayed bolting	cv. Celine (9.8%)	10–62%	21 different mutant alleles	Morineau et al., 2017
		CamV 35S		-	cv. Suneson (15.9%)	~54.7%	A lot of mutant alleles	Jiang et al., 2017
		EC1.2		All *CsFAD2* gene mutants → Stunted bushy phenotype, small, and bloomed late	cv. Suneson (9.8%)	~59.5%	A lot of mutant alleles	Lee et al., 2021
*GhFAD2-1A* *GhFAD2-1D*	CRISPR/Cas9	Ubiquitin (rice)	*Agrobacterium*-mediated transformation	No difference (Fibre quality/length/strength, micronaire, and germination)	13.9%	75.3–77.7%	m1-1 (−41 and +1 bp) m1-2 (+1 and −1 bp) m1-3 (+1 and +1 bp) m20-2 (−1 and +1 bp) m27-3 (−374 bp)	Chen et al., 2021
*TaFAD2*	CRISPR/Cas9	Ubiquitin4-2 (*Petroselinum crispum*)	Floral dipping	Late flowering, shorter plant height, low seed weight per plant, and low germination	12%	~35%	*fad2-4* (−2 bp) *fad2-5* (+1 bp) *fad2-6* (−29 bp)	Jarvis et al., 2021
*OsFAD2-1*	CRISPR/Cas9	Ubiquitin1 (maize)	Biolistic transformation	-	*Oryza sativa Japonica* (No result)	No result	+1 bp -302 bp	Bahariah et al., 2021
		2x 35S	*Agrobacterium*-mediated transformation	No difference	*Oryza sativa cv. Nipponbare* (32%)	~80%	1–1 (+1 bp) 3–11 (+1 bp) 5–17 (−8 bp) 6–23 (−8 bp)	Abe et al., 2018
*GmFAD2*	CRISPR/Cas9	e35S	*Agrobacterium*-mediated transformation	-	cv. JN38 (17.34%)	*GmFAD2-2* (45.08–65.9%)	Substitution, −2, −3, +1, and +2 bp	Al Amin et al., 2019
		tipA	*Agrobacterium*-mediated transformation	No difference in plant height and grain weight. The grain is smaller and deeper in color	cv. JN38 (19.15%)	g3 strain (34.47%) g6 strain (40.45%) g36 strain (72.02%)	*GmFAD2-1A* JN38g3–1 (+1 bp 66.7%) JN38g3–3 (−1 bp 16.6%) JN38g3–4 (−2 bp 16.7%) *GmFAD2-2A* JN38g6–2 (+1 bp 50%) JN38g6–3 (−1 bp 50%) Double JN38g36–3 (+1 and −1 bp 50%) JN38g36–5 (−2 and −7 bp 50%)	Wu et al., 2020
		2x 35S	Hairy root transformation	-	cv. Maverick (~20%)	*GmFAD2-1A, GmFAD2-1B* homozygous lines (~80%)	A lot of mutant alleles	Do et al., 2019

In *Camelina sativa*, which is a hexaploid oil crop, when all three *CsFAD2* were knocked out, oleic acid content increased up to 54–60% but showed a phenotype that did not grow properly in some studies (Jiang et al., 2017; Morineau et al., 2017; Lee et al., 2021). Four *OsFAD2* copies have been identified in rice, and among them, *OsFAD2-1* is most expressed in rice grains (Zaplin et al., 2013). Knockout of *OsFAD2-1* from Japonica with CRISPR/Cas9 did not result in FA analysis (Bahariah et al., 2021), whereas oleic acid levels increased up to 80% in Nipponbare (Abe et al., 2018). *FAD2* of peanut (*Arachis hypogaea*) was also mutated, but the seeds were not harvested; therefore, no FA analysis could be performed (Yuan et al., 2019). In cotton (*Gossypium hirsutum*), it was confirmed that among the eight *FAD2* homologs, *GhFAD2-1A* and *GhFAD2-1D* are mostly expressed in the ovule. Therefore, *GhFAD2-1A* and *GhFAD2-1D* are simultaneously targeted by CRISPR/Cas9. Consequently, the oleic acid content was 75–77% (Chen et al., 2021). In addition, knockout of the *FAD2* gene of pennycress (*Thlaspi arvense*) enhanced oleic acid from 12 to 35% in mutants but delayed the flowering and decreased the germination rate and seed weight (Jarvis et al., 2021).

### Mutation in *FATB* and *KASI*

It is important to reduce the content of SFAs in the food industry because high SFA intake can cause arteriosclerosis in humans (Siri-Tarino et al., 2010). The 16:0-ACP, 18:0-ACP, and 18:1-ACP synthesized from plastids are converted to their free-acyl forms by FATB and FATA which are then released into the cytoplasm and converted into acyl-CoA (Jones et al., 1995; Salas and Ohlrogge, 2002). Knockout of the *FATB* gene through CRISPR/Cas9 has been performed in soybean and peanut (Table 2; Ma et al., 2021; Tang et al., 2022). Soybeans have four GmFATB proteins, all of which have at least 78% homology with *Arabidopsis* FATB at the protein level. *GmFATB2a* and *GmFATB2b* are mainly expressed in flowers, and *GmFATB1a* and *GmFATB1b* are expressed in leaves and seeds. As a result of the simultaneous knockout of *GmFATB1a* and *GmFATB1b* expressed in seeds, the line in which both genes were disrupted showed male sterility. The SFA (palmitic acid and stearic acid) levels of the lines that lost only one of these two genes were 16–21%, but 32.2% in the wild type (Ma et al., 2021). In peanuts, gRNA was designed to target both *AhFATB10a* and *AhFATB10b*, but only a mutation in *AhFATB10a* occurred, which decreased palmitic acid content by approximately 1%, which was slightly lower than that of the wild type (13.3%; Tang et al., 2022).

**Table 2:** Mutation of *FATB*, *KASI* and decrease in the very long chain fatty acid by CRISPR/Cas9.

**Gene name**	**Technique**	**Promoter** **of Cas9**	**Method**	**Phenotype**	**Fatty acid** **WT (%)**	**Fatty acid** **Mutant (%)**	**Mutation type**	**References**
*GmFATB1a* *GmFATB1b*	CRISPR/Cas9	2x 35S	*Agrobacterium*-mediated transformation	*fatb1a*, *fatb1b* (No difference) *fatb1a*:*1b* (Growth defects, male sterility)	SFA (%) (32.28%)	SFA (%) *fatb1a-1* (18.66%) *fatb1a-2* (21.72%) *fatb1b-1* (16.87%) *fatb1b-2* (16.56%) *fatb1a*:*1b* (Male sterility)	*fatb1a-1* (−1 bp) *fatb1a-2* (−1 bp) *fatb1b-1* (−1 bp) *fatb1b-2* (−2 bp) *fatb1a*:*1b* (−1 bp, −30 bp)	Ma et al., 2021
*AtKASI*	CRISPR/Cas9	Ubiquitin	Floral dipping	Smaller and shorter seedlings and semi-dwarf plants	–	–	-54 bp	Xie et al., 2019
*GmKASI*	CRISPR/Cas9	Ubiquitin (soybean)	Whole plant transformation	Homozygous knockout—wrinkled and shriveled seed, increase in sucrose, and decrease in oil content.	cv. Bert 18:2 (49.42–52.56%) 18:3 (7.51–8.55%)	Homozygous mutant 18:2 (30.57–44.56%) 18:3 (15.48–16.99%)	Edit (site1, 2) WPT677-3–35 (+10/+107,WT/WT) WPT677-3–43 (WT/−1,WT/−1) WPT677-3–44 (−1/+1,WT/+1) WPT677-3–48 (WT/−6,WT/+1)	Virdi et al., 2020
*AhFatB10a*	CRISPR/Cas9	Not mentioned	*Agrobacterium*-mediated transformation	No differences	Huayu23 16:0 (13.3%)	16:0 PT1-3 (12.13%) PT1-12 (12.25%) PT1-21 (11.31%) PT2-4 (12.11%) PT2-17 (11.99%)	PT1-3 (1 substitution) PT1-12 (1 substitution) PT1-21 (−2 bp) PT2-4 (1 substitution) PT2-17 (1 substitution)	Tang et al., 2022
*CsFAE1*	CRISPR/Cas9	EC1.1	Floral dipping	No differences compared to Suneson	cv. Suneson20:1 (14.4%)	Less than 1% of 20:1	3–3–1 (−5, −1, and −5 bp) 3–3–3 (−2, −14, and −1 bp) 3–3–4 (−2/+2, −2, and −1 bp) 3–3–14 (−2, −13, and −2 bp)	Ozseyhan et al., 2018
*BnaFAE1*	CRISPR/Cas9	Not metioned	*Agrobacterium*-mediated hypocotyl transformation	Decrease in the seed oil content No differences in agronomic traits	22:1 WH3411 (34.9%) WH3417 (31.0%) GY284 (34.6%)	22:1 WH3411 *c03* (19.3%) *a08c03* (0.07%) WH3417 *c03* (18.8%) *a08c03* (0.03%) GY284 *a08c03* (0.02%)	WH3411 *c03* (−1 bp) *a08c03* (−7/−7 bp) WH3417 *c03* (−2, +1 bp) *a08c03* (−3 bp, 1 substitution/ −2 bp) GY284 *a08c03* (−12 and −2 bp)	Liu et al., 2022
*TaFAE1*	CRISPR/Cas9	Ubiquitin4-2 (*Petroselinum crispum*)	Floral dipping (requires vacuum infiltration)	-	20:1 (15.0%) 22:1 (35.3%)	20:1, 22:1 *fae1-3* (0.9, 0.2%) *fae1-4* (0.9, 0%) *fae1-5* (1.2, 0.1%)	*fae1-3* (−4 bp) *fae1-4* (+1 bp) *fae1-5* (+1 bp)	Mcginn et al., 2019

Among the fatty acid synthases *KASI, II*, and *III* genes, CRISPR/Cas9 was mainly used for *KASI* knockout (Table 2). The in-frame deletion (−54 bp) of *Arabidopsis KASI* causes a semi-dwarf phenotype (Xie et al., 2019). In the *KASI* homozygous mutant of soybean, 18:2 level decreased by 8% and 18:3 level increased by 8.5% compared to the wild-type cultivar Bert. At the same time, the seeds of mutants were wrinkled and shriveled, and the sucrose content increased, while the oil content decreased (Virdi et al., 2020).

### Mutation in *FAE1*

Fatty acids with 12–20 carbons are called long-chain fatty acids (LCFAs), and VLCFAs are longer than 22 carbons (Kihara, 2012). Eicosenoic acid (20:1) and erucic acid (22:1) are produced by the elongation of oleic acid by fatty acid elongase1 (FAE1; Millar and Kunst, 1997). The *Arabidopsis FAE1* gene is mainly expressed in seed embryos (Rossak et al., 2001). Erucic acid, a VLCFA, is associated with myocardial infarction (Imamura et al., 2013). Therefore, researchers have studied the reduction of VLCFA using CRISPR/Cas9 in several plant oils (Table 2).

Simultaneously knocking out three *FAE1* genes in Camelina (cultivar Suneson) decreased erucic acid content to less than 1% (Ozseyhan et al., 2018). In addition, seed weight, oil content, and seed shape were not significantly different from those of “Suneson” (Ozseyhan et al., 2018). In rapeseed, the erucic acid content of the *a08c03* homozygous mutant was reduced to less than 0.1%, and the oil content decreased slightly, but there were no significant differences in other agronomic traits (Liu et al., 2022). In the case of a *c03* single gene mutation, there was no decrease in oil content, and the content of erucic acid was 31–35% in the wild type but decreased by half in the mutant (Liu et al., 2022). In pennycress, the candidate gene of *FAE1* with the highest homology to *Arabidopsis FAE1* was mutated using CRISPR/Cas9. As a result, both 20:1 and 22:1 FAs decreased by less than 1% (Mcginn et al., 2019).

### Mutation in acyltransferases

GPAT, LPAT, and DGAT are acyltransferase enzymes that synthesize TAG by transferring FA from the acyl-CoA pool to G3P (Chapman and Ohlrogge, 2012). In addition, PDAT transfers FA at the *sn-2* position of PC to the *sn-3* position of DAG to synthesize TAG (Dahlqvist et al., 2000). In *Arabidopsis*, there are 10 GPATs, five LPATs, and three DGATs (Zou et al., 1999; Kim and Huang, 2004; Yang et al., 2012; Zhou et al., 2013; Ayme et al., 2018). Among the acyltransferases, GPAT9, LPAT2, DGAT1, and PDAT1 are known to be involved in TAG synthesis (Zou et al., 1999; Banas et al., 2000; Kim et al., 2005; Shockey et al., 2016). Table 3 shows the results of acyltransferase gene editing by CRISPR/Cas9.

**Table 3:** Mutation of acyltransferase and phospholipase by CRISPR/Cas9.

**Gene name**	**Technique**	**Promoter** **of Cas9**	**Method**	**Phenotype**	**Fatty acid** **WT (%)**	**Fatty acid** **Mutant (%)**	**Mutation type**	**References**
*CsDGAT1*	CRISPR/Cas9	CaMV 35S	Floral dipping	Wrinkled and darker seeds, lower oil content	cv. Suneson18:2, 18:3 (22.8, 28.0%)	18:2, 18:3 D4 (25.1, 27.6%) D5 (29.7, 25.8%)	D4,D5 –DGAT1 homozygous mutant	Aznar-Moreno and Durrett, 2017
*CsPDAT1*					Similar to wild type		P1,P3 – PDAT1 homozygous mutant	
*AtGPAT1*	CRISPR/Cas9	CaMV 35S	Floral dipping	Increased the plant height and decreased the seed oil contents Increased the cell length	-	Saturated fatty acids are reduced MUFAs increase	−26 bp	Bai et al., 2021
*BnLPAT2*	CRISPR/Cas9	2x 35S	*Agrobacterium*-mediated hypocotyl transformation	Seed weight decreases, seeds are wrinkled, oil bodies increase	-	Oil content decreases	A lot of mutant alleles	Zhang et al., 2019b
*BnLPAT5*								
*TaROD1*	CRISPR/Cas9	Ubiquitin4-2 (*Petroselinum crispum*)	Floral dipping	No difference	18:1 (12%) 18:2 (18%)	18:1 (~23%) 18:2 (~9%)	*rod1-3* (−18 bp) *rod1-4* (+1 bp) *rod1-5* (+1 bp)	Jarvis et al., 2021
*OsPLDα1*	CRISPR/Cas9	Ubiquitin	*Agrobacterium*-mediated transformation	Phytic acid content Xidao#1 (9.1 mg/g) *osplda1-1* (8.2 mg/g) *osplda1-2*(8.14 mg/g)			*osplda1-1* (−2 bp) *osplda1-2* (−1 bp)	Khan et al., 2019
				Amylose content, pasting properties, and retrogradation properties differ compared to wild type				Khan et al., 2020
*GmpPLA-IIε GmpPLA-IIζ*	CRISPR/Cas9	Not mentioned	*Agrobacterium*-mediated transformation	Knockout mutant is tolerant to iron-deficient condition, droughts, and flooding.			*ppla-IIε/ppla-IIζ-1* (−1 bp, −26 bp) *ppla-IIε/ppla-IIζ-2* (+1 bp, −4 bp) *ppla-IIε/ppla-IIζ-3* (−139 bp, larger –bp and + bp) *ppla-IIε-1* (−14 bp) *ppla-IIε-2* (−4 bp) *ppla-IIζ-1* (−7 bp)	Xiao et al., 2021

As a result of the deletion of *Arabidopsis GPAT1*, SFAs content decreased and MUFAs content increased (Bai et al., 2021). Plant height and cell length increased, but oil content decreased in *gpat1* mutants (Bai et al., 2021). In rapeseed, *Bnlpat2* and *Bnlpat5* single mutants increased the content of 18:0 and 20:0, and decreased the content of 18:1, 18:2, and 18:3 (Zhang et al., 2019b). In the *Bnlpat2 Bnlpat5* double mutant, 20:0 level was increased, while 18:2 and 18:3 levels were decreased. In all these mutants, seed weight decreased, while oil body size increased (Zhang et al., 2019b). In the camelina, *DGAT1* and *PDAT1* were knocked out using CRISPR/Cas9 (Aznar-Moreno and Durrett, 2017). In the *csdgat1* homozygous mutant, 18:2 content was increased and 18:3 content was decreased, and the *cspdat1* homozygous mutant showed an FA composition similar to that of the wild type (Aznar-Moreno and Durrett, 2017). Both mutants showed decreased oil content and the seeds were wrinkled and darkened (Aznar-Moreno and Durrett, 2017). There was no significant change in phenotype when the *REDUCED OLEATE DESATURATION1* (*ROD1*) gene, which interconverts DAG and PC, was mutated in pennycress (Jarvis et al., 2021). FA analysis of mutant seeds showed that 18:1 content was increased and 18:2 content was decreased compared to the wild type (Jarvis et al., 2021).

### Mutation in phospholipases

Phospholipids are plasma membrane lipids (Reszczynska and Hanaka, 2020). Phospholipase is one of the enzymes that hydrolyze phospholipids and is related to various cellular functions (Takac et al., 2019). Plant phospholipases can be categorized as phospholipases A, C, and D (Takac et al., 2019). There are two subtypes of phospholipase A: phospholipase A1 and phospholipase A2. Phospholipase A1 cleaves the acyl group at the *sn-1* position, and phospholipase A2 cleaves the acyl group at the *sn-2* position to release lysophospholipids (LPL; Ryu, 2004). Phospholipase C hydrolyzes phospholipids to release DAG and other phosphorylated head groups (Wang et al., 2012). Phospholipase D cleaves phosphate, releasing its head group and PA (Wang et al., 2012; Figure 1B).

Several studies have been conducted on phospholipase knockout or knockdown through genome editing or RNAi (Table 3; Li et al., 2006; Yamaguchi et al., 2009; Zhao et al., 2011; Guo et al., 2015; Zhang et al., 2019a). In the case of phospholipase research using CRISPR/Cas9, studies have only been conducted on soybeans and rice. In japonica rice cultivar Xidao#1, two independent knockout mutants were generated using CRISPR/Cas9. Analysis of the phytic acid and total phosphorous content of the grain showed a decrease of approximately 9 and 10%, respectively, in the *ospldα1* mutants compared to the wild type (Khan et al., 2019). Additional experiments were performed using the same mutant (Khan et al., 2020). As the LPL content may affect the eating quality of rice, the LPL content in the mutants was checked (Liu et al., 2013). Except for LPC (14:0), the contents of LPC (16:0), LPC (18:1), LPE (14:0), LPE (16:0), and LPE (18:1) were increased by 11–32% in the *ospldα1* mutant compared to the wild type (Khan et al., 2020). These mutants showed a decrease in amylose content, and consequently, low retrograded starch enthalpy, and high gelatinization enthalpy. The pasting property, peak viscosity, hot paste viscosity, breakdown, and cold paste viscosity all increased compared to the wild type, and only the setback viscosity decreased compared with the wild type (Khan et al., 2020). In soybean, three *ppla-Iiε/ppla-Iiζ* homozygous mutants, two *ppla-Iiε* mutants, and one *ppla-Iiζ* mutant were generated and studied (Xiao et al., 2021). Under P-deficient conditions, the main root length was longer in all mutant lines than in the wild type. In the Fe-deficient condition, all mutants had higher chlorophyll content, although there was a slight difference between mutants. The most shoot and root fresh weights of mutants were the same or higher than those of the wild type (Xiao et al., 2021).

### Mutation in TAG lipases

Triacylglycerol lipase catalyzes the hydrolysis of TAG to release G3P and FAs (Graham, 2008). TAGs are stored and mobilized in the form of lipid droplets (LDs), and oleosins play a role in maintaining the LD structure (Huang, 2018). In *Arabidopsis*, TAG is degraded by *SUGAR DEPENDENT 1* (*SDP1*) to release FAs (Eastmond, 2006). In the *sdp1* mutant, it was identified that SDP1-LIKE (SDP1L) has a function to hydrolyze the TAG (Kelly et al., 2011). In addition to SDP1, OIL BODY LIPASE 1 (OBL1) was discovered in castor (*Ricinus communis*) and it can hydrolyze the TAG (Eastmond, 2004). The *OBL1* gene was also identified in Arabidopsis and tobacco, both of which are located in lipid droplets (LDs) and play an important role in pollen tube growth (Muller and Ischebeck, 2018). AtOBL1 has a lipase activity to TAG, DAG, and 1-MAG (Muller and Ischebeck, 2018). After the lipase degrades the TAG, FAs enter the peroxisome through *PEROXISOMAL ABC-TRANSPORTER1* (*PXA1*), where the beta-oxidation process occurs, in which carbon is broken down by two to form acetyl-CoA (Poirier et al., 2006). Acetyl-CoA enters the TCA cycle to generate energy sources, such as ATP, NADH, and FADH2 (Martinez-Reyes and Chandel, 2020). Disruption of TAG lipase using CRISPR/Cas9 has not been attempted in various plants. However, it has been reported that *SDP1* is disrupted using RNAi technology (Table 4; Kelly et al., 2013; Kim et al., 2014; Kanai et al., 2019; Azeez et al., 2022; Aznar-Moreno et al., 2022). When the *SDP1* expression in *rapeseed* was decreased by RNAi, the oil yield (g/ $m^{2}$ ) was further increased by 8% without affecting fatty acid composition (Kelly et al., 2013). The germination, shoot growth, and root growth are unaffected although the germination rate of seeds harvested 2 years ago decreased slightly (Kelly et al., 2013). When the *SDP1* gene in *Jatropha curcas* was knockdown, the total lipid content of endosperm was increased compared to the control, but there was no significant difference in fatty acid composition (Kim et al., 2014). The knockdown of four *GmSDP1* increased the seed weight, seed yield, and oil yield (g/plant), and oleic acid content was increased whereas linoleic acid content was decreased (Kanai et al., 2019). *GmSDP1* was targeted by RNAi, which lead to enhance seed weight and overall lipid content but decreased the content of raffinose family oligosaccharides (Aznar-Moreno et al., 2022). Seed-specific silencing of the *SDP1* gene in *Physaria fendleri* by RNAi increased seed weight and lipid content with the normal seedling establishment except for one line (Azeez et al., 2022). Based on previous results, it is possible to increase the oil content by knocking out TAG lipase using CRISPR/Cas9. Lipase has also been related to oil rancidity (Bhunia et al., 2021; Kumar et al., 2021). Rice bran oil (RBO) is abundant in nutrients but rapidly becomes rancid (Raghuram and Rukmini, 1995; Bhunia et al., 2021). Pearl millet seeds also have a high nutrition quality but the flour goes rancid rapidly (Kumar et al., 2021). Even though TAG is broken down by lipase and used as an energy source for germination, many fatty acids are released, which adversely affects rancidity (Kumar et al., 2021). The putative lipases were identified in rice and pearl millet (Bhunia et al., 2021; Kumar et al., 2021). In pearl millet, *PgTAGLip1* and *PgTAGLip2* polymorphisms were identified to cause loss-of-function mutation in an inbred line that low rancidity (Aher et al., 2022). Therefore, the disruption of lipase by CRISPR/Cas9 in rice or pear millet may be a key point in avoiding rancidity (Bhunia et al., 2021; Kumar et al., 2021).

**Table 4:** TAG lipase and increasing of TAG in vegetative tissues.

**Gene name**	**Technique**	**Method**	**Promoter**	**Phenotype in transgenic**	**Oil content** **(WT)**	**Oil content (Transgenic)**	**References**
*BnaSDP1* (GN078283)	RNAi	*Agrobacterium*-mediated transformation	USP (from *Vicia faba*)	No difference in FA composition Little adverse impact on seed vigour	cv. Kumily 42.36 ± 0.12%	43.84 ± 0.10 ~ 45.86 ± 0.13%	Kelly et al., 2013
*JcSDP1*	RNAi	Electroporation	*JcSDP1*	No difference in seed size	-	Increased the total lipid content in endosperm (% w/w)	Kim et al., 2014
*GmSDP1-1* *GmSDP1-2* *GmSDP1-3* *GmSDP1-4*	RNAi	*Agrobacterium*-mediated transformation	Soybean 11S globulin	Rupture of seed coat Increased the seed weight (g/seed) Increased the 18:1 but no difference in 16:0, 18:0 and 18:3	Kariyutaka	Increased the seed yield (g/plant), oil yield (g/plant)	Kanai et al., 2019
*GmSDP1-1* *GmSDP1-2*	RNAi	*Agrobacterium*-mediated transformation	Soybean glycinin	Seed weight of mutants are ranging from 208 to 226 mg/seed (WT-183 mg/seed) Increased the total lipid (mg/seed) Germination rate is slower than WT	Williams82 (23.3%)	Fatty acid content (24.3%)	Aznar-Moreno et al., 2022
*GmSDP1-3* *GmSDP1-4*						Fatty acid content (24.4%)	
*PfrSDP1*	RNAi	*Agrobacterium*-mediated transformation	2S albumin	Seed weight of mutants are ranging from 0.74 to 0.77 mg (WT-0.66 mg)	Lipid content (228 μg per mg seed) 20:1-OH, 20:2-OH (122.2 and 7 μg per mg seed)	Lipid content (261–271 μg per mg seed) 20:1-OH, 20:2-OH (144.8–155 and 8.9–10.8 μg per mg seed)	Azeez et al., 2022
*AtDGAT1*	Overexpression	Leaf-disc *Agrobacterium*-mediated technique	*rbcS*	Decrease the amount of 18:3 and increase the amount of 18:1	Total FA content (2.8%)	Total FA content (~5.6%)	Andrianov et al., 2010
*AtLEC2*			*Alc*		FA content (2.9% of dry weight)	FA content (6.8% of dry weight)	
*AtDGAT1*	Overexpression	Leaf-disc *Agrobacterium*-mediated technique	*PtdCesA8A*	Minor difference about number of branches and stem diameter Increased the amount of 18:1, 18:2, and 18:3 in stems	-	Increased the oil bodies in pith, xylem, and cortex tissues Increased the total FA and TAG content	Nookaraju et al., 2014
*AtLEC2*							
*AtDGAT1* *AtWRI1* *SiOLEOSIN*	Overexpression	*Agrobacterium*-mediated transformation	CaMV 35S, RuBisCO small subunit	No negative phenotype of development Increased the amount of 18:1, 18:2 and decreased the amount of 18:3 in leaves	TAG content (% DW) (~0.2%)	TAG content (% DW) (~15.8%)	Vanhercke et al., 2014
*NtSDP1*	RNAi	*Agrobacterium*-mediated transformation in transgenic lines (Vanhercke et al., 2014)	enTCUP2	Reduction of starch content	TAG content (% DW) (0.1%)	TAG content (% DW) (~29.8%)	Vanhercke et al., 2017
*AtLEC2*	Overexpression		SAG12			TAG content (% DW) (~33.3%)	
*VgDGAT1a*	Overexpression	Transient expression	CaMV 35S	No difference of plant morphology but change the tuber morphology, germination rate and leaf chlorophyll content Increased the 18:2 and decreased the 18:3	-	TAG content (% DW) (~9.2%)	Gao et al., 2018
*NtAn1*	CRISPR/Cas9	Leaf-disc Agrobacterium-mediated technique	2x 35S	Yellow seed coat and white flower Decreased the PAs content and stearic acid No difference of seed size, seed weight and seed number per fruit	Lipid content (38.77 μg/seed)	Lipid content (44.97-45.91 μg/seed)	Tian et al., 2020b
*AtACC1*	Overexpression	Leaf-disc Agrobacterium-mediated technique	CaMV 35S	Increased the amount of 18:2 and decreased the amount of 18:3	Relative amount of TAG (1.2 mol %) Total FA content (1.08 mg/g FW)	Relative amount of TAG (4.6 mol %) Total FA content (1.35–1.39 mg/g FW)	Klaus et al., 2004
*AtWRI1*	Overexpression	Agrobacterium-mediated transformation	GBSS	No difference of plant morphology but change the tuber morphology Increased the amount of 18:2 and decreased the amount of 18:3	-	Increase the TAG and polar lipid (nmol FA/mg DW)	Hofvander et al., 2016
*AtDGAT1* *AtWRI1* *SiOLEOSIN*	Overexpression	Agrobacterium-mediated transformation	CaMV 35S, patatin class I promoter B33	Increased the soluble sugar content and decrease the starch content Decreased the SFA and 18:3 but increase the MUFA in tuber	TAG content (% DW) (0.03%)	TAG content (% DW) (~3.3%) Increase the polar lipid	Liu et al., 2017
*StAGPase* *StSDP1*	RNAi	Electroporation	CaMV 35S	Incraease the total sugar content and decrease the total starch content in mature potato tuber	Total FA content in mature potato tuber (0.3%)	Total FA content in mature potato tuber (~2.95%)	Xu et al., 2019

## Increase the TAG in vegetative tissue

In addition to plant seeds, TAG content can be increased in vegetative tissues such as leaf and tuber (Xu et al., 2018). In tobacco, oil enhancement is mainly achieved by overexpression of DGAT1 or positive transcription factors such as LEAFY COTYLEDON2 (LEC2) and WRINKLED1 (WRI1) (Table 4; Andrianov et al., 2010; Nookaraju et al., 2014; Vanhercke et al., 2014, 2017; Gao et al., 2018). TAG content was enhanced 20-fold in tobacco leaves when *AtDGAT1* was expressed under the control of the ribulose-biphosphate carboxylase small subunit promoter (Andrianov et al., 2010). *AtLEC2* was expressed under the control of the inducible *Alc* promoter (Andrianov et al., 2010). As a result, the FA content increased from 2.9% up to 6.8% (per dry weight) when treated with 1% acetaldehyde (Andrianov et al., 2010). Arabidopsis *DGAT1* or *LEC2* expression driven by the xylem-specific promoter in tobacco increases the FA and TAG content in the stem (Nookaraju et al., 2014). *AtWRI1*, *AtDGAT1*, and *Sesamum indicum OLEOSIN* (*SiOLEOSIN*) genes were transformed simultaneously into tobacco, and 15.8% of TAG was found in tobacco leaves (Vanhercke et al., 2014). *AtLEC2* overexpression or silencing of *SDP1* in transgenic tobacco (Vanhercke et al., 2014) accumulated the TAG up to 29.8 and 33.3% in the leaves, respectively (Vanhercke et al., 2017). Overexpression *DGAT1a* from *Vernonia galamensis* L. in tobacco, the TAG content of the leaves was enhanced up to 9.2% (per dry weight) without any deleterious phenotype (Gao et al., 2018). Two transgenic lines were generated using CRISPR/Cas9 by knocking out the *NtAN1* gene, which regulates proanthocyanidins (PAs) and lipid accumulation in tobacco (Tian et al., 2020b). These mutants enhanced the lipid and protein content and also displayed yellow seed coat (Tian et al., 2020b).

Triacylglycerol enhancement research in the potato (*Solanum tuberosum*) tuber was also conducted (Table 4; Klaus et al., 2004; Hofvander et al., 2016; Liu et al., 2017; Xu et al., 2019). Arabidopsis *ACCase* was expressed in potato, the FA increased by 30% relative to the wild type, and showed a 5-fold increase in TAG accumulation compared to that of the wild type (Klaus et al., 2004). *AtWRI1* was expressed under the control of the *GBSS* promoter. TAG increased 20-fold compared to that of wild type, and the polar lipid was also increased (Hofvander et al., 2016). Three genes were expressed simultaneously in the potato: *AtDGAT1*, *AtWRI1*, and *SiOLEOSIN* controlled by the 35S and B33 potato patatin promoters (Liu et al., 2017). As a result, TAG, which was 0.03% in the wild type, was enhanced up to 3.3% in the tuber (Liu et al., 2017). When potato *ADP-glucose pyrophosphorylase* (*AGPase*) and *SDP1* were simultaneously silenced using RNAi, TAG content in the mature tuber of potato was increased by 16-fold compared to that of the wild type (Xu et al., 2019). TAG enhancement in vegetative tissue was mainly caused by overexpression or knockdown in tobacco and potato. In the future, if CRISPR is applied to enhance the TAG through lipid gene editing of tobacco or potato, it will be regarded as an important biofuel platform.

## Conclusion and future perspective

Fatty acids constitute TAG, an energy source as well as a component of cell membranes and chloroplast membranes that are essential for plant cells (Kim, 2020). Since TAG in plant oil is a major source of food and industrial raw materials, attention has been focused on changing the FA composition and increasing the TAG content (Xu and Shanklin, 2016). So far, research has focused on creating high-oleic acid plant varieties by removing the FAD2 function (Table 1). Lately, research is underway to develop high-oleic acid varieties using multiple gRNAs to target both *FAD2* and *FATB* genes in soybean (Kim et al., 2021). In rapeseed, researchers used CRISPR/Cas9 to target *FAE1* and diminish the levels of 20:1 and 22:1 (Table 2). To lower the 20:1 and 22:1 content while further increasing the 18:1 content, *FAD2* of pennycress was mutated in the *fae1* knockout background (Jarvis et al., 2021). Moreover, CRISPR/Cas9 is commonly used in crops to investigate the roles of acyltransferase, phospholipase, and FAS genes (Tables 2, 3).

**Figure 3:**
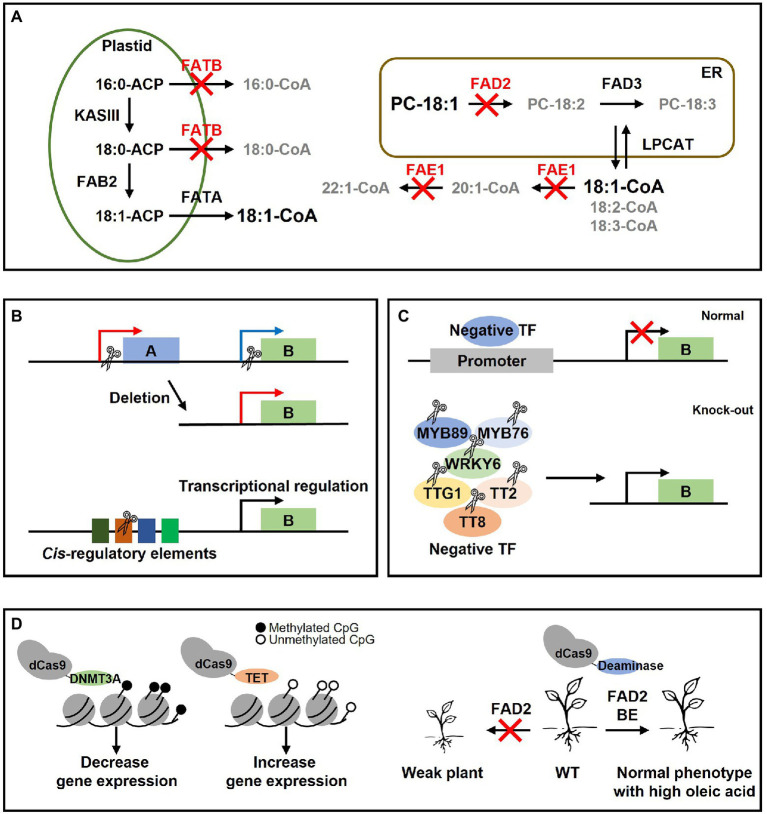
Future strategies of lipid metabolism research using CRISPR/Cas9 in this paper. **(A)** Schematic diagram of fatty acid synthesis for the development of plants with high oleic acid content in seeds. The flow of fatty acid synthesis is indicated by arrows. Red letters indicate three genes that may increase the oleic acid content if it is eliminated by CRISPR. Gray letters indicate fatty acids whose content is decreased when three genes (*FATB*, *FAE1*, and *FAD2*) are knocked out. It appears that plants with high oleic acid content may be created if three genes were deleted. **(B)** A study of promoter regulation using CRISPR/Cas9. B refers to the lipid gene and A is the upstream gene of the B gene. The scissor shape represents CRISPR/Cas9. By removing from the 5′UTR of the B gene to the 5′UTR of the A gene, the B gene can be controlled by the A promoter (Bhunia et al., 2022). In addition, the transcription level can be regulated by deleting the *cis*-regulatory elements of the B gene. **(C)** Knockout of the negative transcription factor in plant. The expression of lipid genes can be reduced in a normal plant by a variety of negative transcription factors. However, if the negative transcription factor is disrupted using CRISPR, the expression of the lipid gene can be increased. **(D)** CRISPR/Cas9-based technology. Epigenetic study of lipid gene seems possible if Cas9-based technique is used. For example, by methylation through dCas9-DNMT3A, lipid gene expression can be suppressed whereas demethylation *via* dCas9-TET can increase lipid gene expression. Alternatively, it is possible to develop a plant that slightly weakens the function of a specific protein by using base editing and has a normal phenotype than that of knockout mutants, but with a changed lipid composition.

For future applications of CRISPR/Cas9 to lipid metabolism research, we suggest four possible strategies. First, CRISPR/Cas9 can be used to abolish the function of multiple lipid metabolism genes. Crops with high oleic acid content can be developed by simultaneously knocking out the *FAE1* gene, which elongates the 18:1 to 20:1, 22:1, and the *FAD2* gene, which desaturases the 18:1 to 18:2. Alternatively, it seems possible to develop crops with high oleic acid if *FATB* gene is mutated in *fad2* or *fae1* mutants (Figure 3A). Using CRISPR in mutants in which a specific gene has already been disrupted by EMS or RNAi may be a good strategy. For example, if *fad2* is mutated using CRISPR/Cas9 in the *fae1* EMS mutant line (Ozseyhan et al., 2018), a Camelina with higher oleic acid levels can also be developed. However, if there has no mutant background in which the lipid metabolism gene was disrupted by EMS or RNAi, multiple gRNAs can be used to target each distinct gene at once to abolish numerous gene functions simultaneously. Second, expression of the target gene can be controlled by removing the whole promoter region or *cis*-regulatory elements (CREs) using CRISPR/Cas9 (Figure 3B). For example, *DGAT2 UPSTREAM GENE 1* (*DUG1*) which exists upstream of *DGAT2* has a higher expression in leaves than *DGAT2*. Therefore, they deleted the 5′UTR region of *DUG1* to the 5′UTR region of *DGAT2* in Arabidopsis *sdp1* mutant using CRISPR/Cas9 so that *DGAT2* was controlled by the *DUG1* promoter (Bhunia et al., 2022). As a result, total lipid content (% cell dry weight) in leaves increased 2-fold and TAG content (% CDW) increased 30-fold compared to *sdp1* mutant (Bhunia et al., 2022). This method can be used only if the promoter direction of the upstream gene is appropriate (Bhunia et al., 2022). We think that the function of the upstream gene is irrelevant to plants since it will be eliminated by CRISPR/Cas9. It is also important to investigate the promoter expression level or tissue-specific expression of the upstream gene in advance. Deletion of CREs using CRISPR/Cas9 may be an effective strategy for regulating the transcription level of lipid genes (Figure 3B). Knockout causes complete loss of gene function, whereas deletion of CREs allows to fine-tune desirable traits more than knockout (Wolter et al., 2019). In fact, research was performed on the development of plants with agriculturally good traits by eliminating CREs using CRISPR/Cas9 (Li et al., 2020a, 2022; Wu et al., 2021). Third, it may be necessary to eliminate negative transcription factors that regulate the expression of lipid metabolism genes using CRISPR/Cas9 (Figure 3C). WRI1 (Baud et al., 2009), LEC1 (Mu et al., 2008), LEC2 (Kim et al., 2015), MYB96 (Lee et al., 2018), BASIC LEUCINE ZIPPER TF 67 (bZIP67; Mendes et al., 2013; Kim et al., 2022), ABSCISIC ACID INSENSITIVE 3 (ABI3; Giraudat et al., 1992), FUSCA3 (FUS3; Luerssen et al., 1998) have been reported as positive regulators of TAG biosynthesis. MYB89 (Li et al., 2017), WRKY6 (Song et al., 2020), MYB76 (Duan et al., 2017), TRANSPARENT TESTA GLABRA1 (TTG1; Chen et al., 2015), TRANSPARENT TESTA2 (TT2; Chen et al., 2012), and TT8 (Chen et al., 2014) have been reported as negative regulators. Therefore, knockout of the negative regulator using CRISPR/Cas9 in various oil crops may enhance the oil content. It is important to choose a negative TF that only increases oil content without any detrimental growth phenotype when the negative TF is eliminated because transcription factors can regulate not only lipid-related genes but also other genes. Finally, CRISPR/Cas9-based methods can be applied in plants by using dCas9 as a carrier, which is an inactive Cas9 (Figure 3D). Recently, DNMT3A, which methylates DNA, was fused with dCas9 to methylate DNA at a specific site to decrease gene expression, or TET was fused with dCas9 to demethylate DNA to increase gene expression (Xie et al., 2018). These techniques will be a new approach in the epigenetic study of genes involved in lipid metabolism. In addition, the base editor can be a good strategy to change one amino acid or mutate randomly in the plant genome (Figure 3D). For example, with random mutation of the *FAD2* gene of *Arabidopsis* using base editing, the activity of FAD2 was weakened, and individuals with high oleic acid and resistance to salt stress were selected (Park et al., 2021).

There is a point to note when CRISPR/Cas9 is applied to mutate some genes. This is because mutations in lipid metabolism genes can alter lipid composition while also adversely affecting plant growth and development. For example, *Camelina* has three copies of the *FAD2* gene because it is allohexaploid (Kang et al., 2011). If all three copy *FAD2* genes are completely mutated, it adversely affects plant growth because this mutant is unable to synthesize polyunsaturated FAs, which are essential for maintaining the fluidity of cell membranes (Lee et al., 2021). To avoid such extreme phenotypes, it is necessary to specifically knockout only one or two *FAD2* genes in camelina (Lee et al., 2021). In addition, in plants with a single copy of *FAD2*, it may be preferable to create a weak allele using the base editor (Park et al., 2021). A potential problem is that Cas9 can bind to unintended sites causing accidental mutations, or off-target mutations. However, these off-target mutations can be overcome in plants by backcrossing with wild type. In the future, oil crops that produce a large amount of useful FA for the industry should be developed by simultaneously controlling transcription factors and lipid metabolic genes using genome editing.

## Author contributions

M-EP and HUK designed and structured the review, collected the information, organized the figures and tables, and wrote and revised the manuscript. All authors contributed to the article and approved the submitted version.

## Funding

This work was supported by grants from the Mid-Career Researcher Program of the National Research Foundation of Korea (NRF-2020R1A2C2008175), the New Breeding Technologies Development Program (project no. PJ016533), and the Next Generation BioGreen21 associated program (project no. PJ015714), and the Rural Development Administration, Republic of Korea.

## Conflict of interest

The authors declare that the research was conducted in the absence of any commercial or financial relationships that could be construed as a potential conflict of interest.

## Publisher’s note

All claims expressed in this article are solely those of the authors and do not necessarily represent those of their affiliated organizations, or those of the publisher, the editors and the reviewers. Any product that may be evaluated in this article, or claim that may be made by its manufacturer, is not guaranteed or endorsed by the publisher.
